# The sensory-motor theory of rhythm and beat induction 20 years on: a new synthesis and future perspectives

**DOI:** 10.3389/fnhum.2015.00444

**Published:** 2015-08-26

**Authors:** Neil P. M. Todd, Christopher S. Lee

**Affiliations:** ^1^Faculty of Life Science, University of ManchesterManchester, UK; ^2^Department of Psychology, Goldsmiths College, University of LondonLondon, UK

**Keywords:** rhythm perception, beat induction, vestibular system, sensory-motor integration, music and movement, auditory cortex

## Abstract

Some 20 years ago Todd and colleagues proposed that rhythm perception is mediated by the conjunction of a sensory representation of the auditory input and a motor representation of the body (Todd, 1994a, 1995), and that a sense of motion from sound is mediated by the vestibular system (Todd, 1992a, 1993b). These ideas were developed into a sensory-motor theory of rhythm and beat induction (Todd et al., 1999). A neurological substrate was proposed which might form the biological basis of the theory (Todd et al., 2002). The theory was implemented as a computational model and a number of experiments conducted to test it. In the following time there have been several key developments. One is the demonstration that the vestibular system is primal to rhythm perception, and in related work several experiments have provided further evidence that rhythm perception is body dependent. Another is independent advances in imaging, which have revealed the brain areas associated with both vestibular processing and rhythm perception. A third is the finding that vestibular receptors contribute to auditory evoked potentials (Todd et al., 2014a,b). These behavioral and neurobiological developments demand a theoretical overview which could provide a new synthesis over the domain of rhythm perception. In this paper we suggest four propositions as the basis for such a synthesis. (1) Rhythm perception is a form of vestibular perception; (2) Rhythm perception evokes both external and internal guidance of somatotopic representations; (3) A link from the limbic system to the internal guidance pathway mediates the “dance habit”; (4) The vestibular reward mechanism is innate. The new synthesis provides an explanation for a number of phenomena not often considered by rhythm researchers. We discuss these along with possible computational implementations and alternative models and propose a number of new directions for future research.

Πάντα ῥεĩ

–*Heraclites (c 540 – 475 BC)*

## 1. Historical background

### 1.1. Music and movement in antiquity and in contemporary writing

As has been described previously (e.g., see Fraisse, 1982 or Todd, 1995), the idea of a link between music and movement is one which can be traced back to antiquity and prehistory. It would be impossible to do justice here to the history of this idea and its development within the constraints of the present format. However, it is useful to provide at least a brief review. It has been suggested that within the classics, notions of music and movement can be found in the writings of Heraclites, as may be inferred from the aphorism quoted above meaning “everything flows” (Brandner, 2012). Such concepts are clearly established by the time of Aristotle. Aristoxenus of Tarentum, a pupil of Aristotle, in his Elements of Rhythm (*Elementa Rhythmica*) (c 300 BC), compared the movement in speaking and singing to the body making a gesture or dancing. For Aristides Quintilianus in his work *On Music* (Περί Moυσικῆç) (c 300 AD), sound and movement formed the very fabric of music. Both Aristoxenus and Aristides could be said to belong to an Aristotelian school of thought in emphasizing change rather than the more Pythagorean ideal of ratios (Barker, 1989). Such notions have been recapitulated throughout the history of writing on music and became embedded in the very language used in the practice of music, especially expression markings used in musical notation. Tempo markings can be seen from the ninth century, but additional expression terms blossomed in the Sixteenth and Seventeenth centuries which were strongly linked to dance style (Encyclopedia Britanica, 2014).

In more scientific literature from the Nineteenth century, for Von Helmholtz motion in music was intimately linked to emotion (Helmholtz, 1885). Stetson (1905) in his motor theory of rhythm drew a link between gesture in movement and phrasing in music. In the music psychology literature of the Twentieth century, motion features strongly in Seashore's (1938) book. However, it is in the work of Seashore's contemporary Truslit (1938) *Gestaltung und Bewegung in der Musik* that the link between music and motion is given a fully articulated theory (Repp, 1993; Brandner, 2012). Truslit's approach was strongly informed by a school of piano practice emphasizing gesture in performance, which can be traced back to Liszt and included Truslit's teacher Elizabeth Caland. In this period there was in society a broader music and movement current, prominent within which was the dancer Isadora Duncan (Brandner, 2012). Among the ideas within Truslit's writings was the hypothesis that the vestibular system must have a role. In building a case for a vestibular link Truslit cited a variety of evidence including the work of Tullio (1929), a name which is well-known in contemporary vestibular medicine (Watson et al., 2000). As pointed out by Repp (1993), there are many affinities with work of the later Twentieth century by Clynes (1977), Gabrielsson et al. (1983), and Gabrielsson (1987).

It is perhaps in the work on expression in musical performance that the motional concepts came to the fore most prominently in contemporary work dating from the 1980s. Todd (1985) modeled expressive timing in piano performance on a motion gesture and then later revised and extended the model to introduce an explicit motional formulation (Todd, 1992a, 1995). During this period several authors also produced analytic or synthetic models of expression which made a direct link to physical or biological motion (Sundberg and Verrillo, 1980; Kronman and Sundberg, 1987; Longuet-Higgins and Lisle, 1989; Feldman et al., 1992; Shove and Repp, 1995; Friberg and Sundberg, 1999; Gabrielsson, 1999).

### 1.2. Motion from sound, the amplitude modulation spectrometer

**Figure 1:**
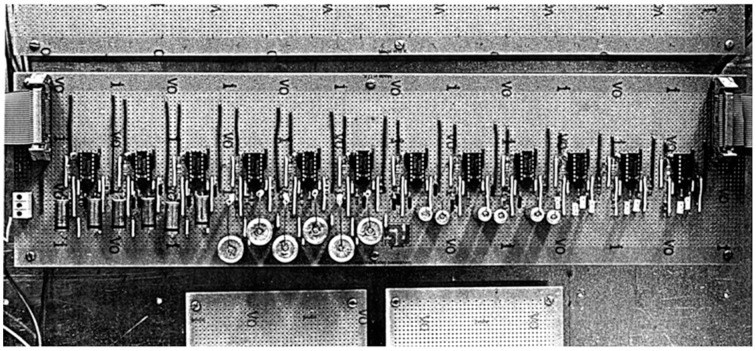
**The first filter-bank rhythm analyzer (Todd, 1993a)**. The original amplitude modulation spectrometer was implemented as an analog system using biquad circuits over the range 0.125–16 Hz. Each circuit simultaneously gave a low-pass and band-pass output. It was constructed at City University, Music Department during 1991/2, sadly later destroyed during a flood at Sheffield University Music Department.

An important concept to emerge from this work was another Truslitian notion that during a musical performance a performer is attempting to communicate a sense of motion and that rhythmic structure and motion are two sides of the same coin, so to speak. This then raised the question of how a listener could recover structure from motion in sound. What perceptual mechanisms could be at play? It was at this time that Truslit's vestibular hypothesis was revived independently by Todd. In an early attempt to capture this notion in a model a simple filter approach was advocated (Todd, 1991, 1992a,b). A good way of thinking about this is that rhythmic communication is a bit like AM radio, except that the carrier wave is audio and the signal is the rhythmic amplitude changes associated with movement. However, since rhythmic motion structure is hierarchical it was necessary to propose that the perceptual system makes use of a bank of such filters with a range of time-constants. Thus, was born the rhythmic modulation spectrometer (Todd, 1993a) (see Figure 1).

Initially implemented as an analog system the core of the analyser was a dual constant-Q low-pass/band-pass filter-bank logarithmically spaced over the range approximately 0.125–16 Hz, which corresponds to the range of movement frequencies found in a rhythmic structure. When connected to an analog recorder it could drive the ink-pens in real time to produce an analysis of a communicative sound structure of any description, be it human music or poetry, or bioacoustic signals such as bird song or insect chirps. For very expressive music, such as the piano works of Chopin, it could capture the motion structure at each level from the low-pass output. For regular rhythms with a clear beat it could also extract a beat from the band-pass output.

### 1.3. The sensory-motor theory emerges

The invention of the analog AM spectrometer or rhythm analyser was a useful step in enabling the visualization of motion/rhythm structure from sound and in formulating for the first time the precise specification of the parameters involved in carrying out such an analysis. However, it also raised a number of issues and problems. One immediate issue was that an analog implementation lacks flexibility. A digital version of the filter-bank was implemented, which was relatively trivial to accomplish with suitable signal-processing methods, but more fundamental issues also arose.

**Figure 2:**
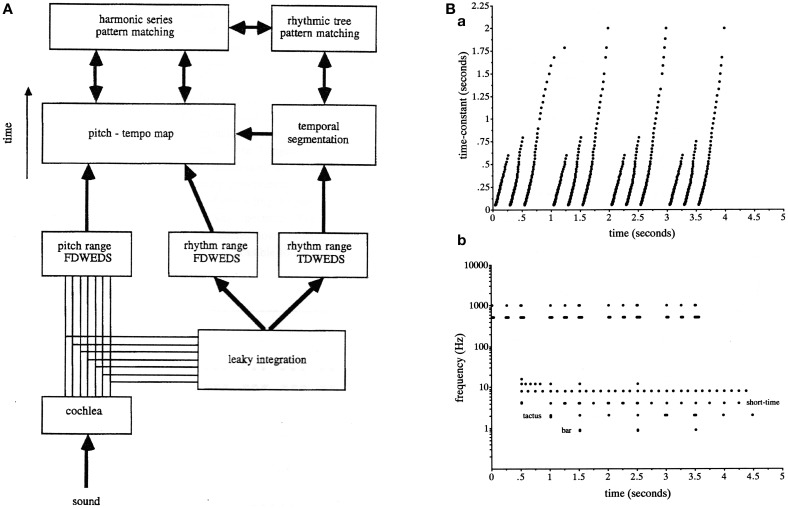
**(A)** A reproduction of Figure 16 from Todd (1994a) illustrating the scheme for computing a modulation spectrum in both pitch and rhythm frequency ranges and in parallel both low-pass and band-pass representations. The abbreviation FDWEDS stands for “frequency domain windowed energy density spectrum” (i.e., the output of a band-pass filter bank). The abbreviation TDWEDS stands for “time domain windowed energy density spectrum” (i.e., the output of low pass filter bank). **(B)** A reproduction of Figure 17 from Todd (1995) illustrating low-pass vs. band-pass responses which compute approximately the grouping and metrical structure of an anapest rhythm. **(B)** shows an output of the scheme represented in **(A)**.

The first was that because of the limitations defined by the uncertainty principle in the simultaneous location of events in time and their rate (inverse period) it appeared necessary to separate the low-pass and band-pass filters into two distinct analyses (Todd, 1994b). Figure 2A reproduces from Todd (1994a) the overall computational scheme that was employed at this time. After analysis with an ear model front end, separate filter-banks were used in parallel. Both were constant-Q and logarithmically spaced in frequency, i.e., computed a wavelet rather than a Fourier transform of the AM signal. The low-pass output it was suggested was good for locating events in time, whereas the band-pass output was good for locating periods between events, thus overcoming the uncertainty principle limitations on measurement of time and frequency in a single filter (see Figure 2B).

In an attempt to place the AM filter approach to audio-temporal processing in a more biological context, i.e., consistent with auditory neuroanatomy and physiology, a theory of the central auditory system was developed subsequently which integrated timbre, pitch and rhythm into a single description (Todd, 1996b,c). The essential idea was that temporal information is spatially coded in three dimensions, corresponding approximately with the cochlea, the inferior colliculus and cortex. In this scheme periodicity pitch was primarily associated with sub-cortical processing, creating a stabilized auditory image, whereas time and rhythm were associated with auditory cortex, processing rhythmic movement or flow within the image. Such a scheme might be realized by populations of auditory cortical spectro-temporal receptive fields tuned to different features. This theory of the central auditory system, it was argued, could account for a variety of pitch and rhythmic phenomena (Todd, 1996a,b,c).

**Figure 3:**
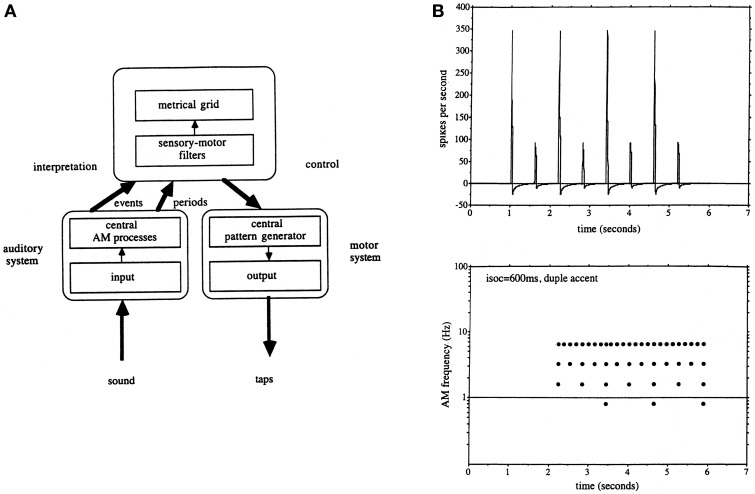
**(A)** A reproduction of Figure 12 from Todd and Brown (1996) illustrating the auditory-motor hypothesis as it was envisaged at this time. The auditory system computed both low-pass and band-pass representations of a rhythmic sequence. This then allowed a central process to make use of a motor representation to interpret the meter and select an appropriate metrical grid. **(B)** A reproduction of Figure 16 from Todd and Brown (1996) illustrating a simulated auditory nerve input and metrical grid response to a duple accent rhythm.

The second fundamental issue which arose from the original modulation spectrometer was that in order to account for beat detection it was clear that a band-pass filter-bank was not sufficient on its own since temporal intervals were represented in the form of a harmonic series, and there was no apriori way of selecting which harmonic should be the tactus, although the choices could be reduced by harmonic series pattern matching. It was at this point therefore, noticing the coincidence of tactus and locomotion rates, a link which had been suggested many times previously (e.g., see Fraisse, 1982), that Todd and colleagues evoked the idea of a motor component as a way of selecting a metrical harmonic, and the audio-motor hypothesis first appeared (Todd and Lee, 1994; Todd, 1995; Todd and Brown, 1996). Figure 3 reproduces the signal-processing scheme envisaged in Todd and Brown (1996) and a sample metrical analysis. In this scheme a metrical harmonic was selected by means of a sensory-motor filter, or internal representation of the dynamics of the motor system, a concept similar to Jackendoff's (1988) internal body representations which mediate the perception of dance. This was then used to drive an output. An important question which subsequently presented itself was if rhythm and beat induction was mediated by an audio-motor interaction, how could this work?

**Figure 4:**
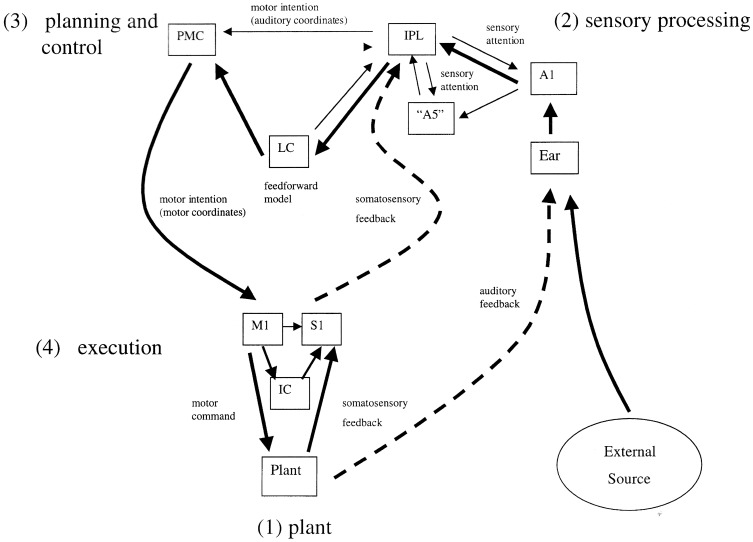
**A reproduction of Figure 2 from Todd et al. (2002) illustrating the principal brain representations and their connections according to the neurobiological theory of temporal tracking and beat induction**. (1) The plant, (2) the sensory processing system, (3) the control and planning system, and (4) the motor execution system (A1 primary auditory cortex, “A5” secondary auditory cortex; IPL, inferior parietal lobule; PMC, premotor cortex; LC, lateral cerebellum; M1, primary motor cortex; S1, primary somatosensory cortex; IC, intermediate cerebellum).

**Figure 5:**
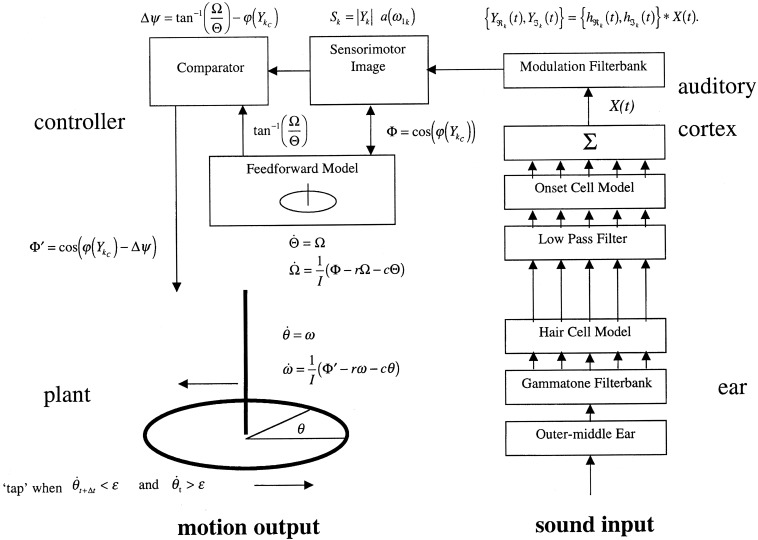
**A reproduction of Figure 3 from Todd et al. (2002) illustrating the principal elements of a computational algorithm which implements the neurobiological theory**. The system is represented operating in open-loop mode, i.e., without feedback.

**Figure 6:**
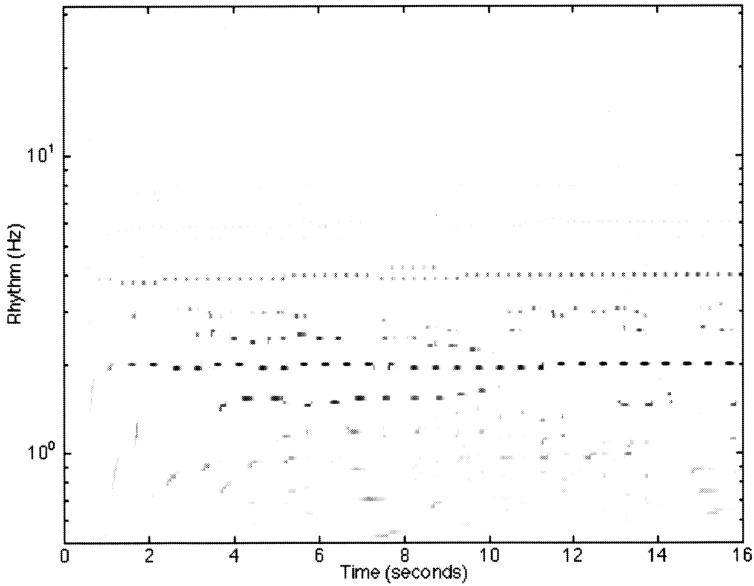
**A reproduction of Figure 12 from Todd et al. (1999) illustrating the model response to the first 4 bars of the Fugue No. 2, Bk I of the Well-Tempered Clavier by JS Bach**. The sampling rate of the input was at 22,050 Hz with 8 bit resolution. Thirty-two cochlear channels were used ranging from 30 Hz to 8000 kHz. All cortical filters ran at a sampling rate of 1000 Hz. The frequencies of the cortical band-pass filters ranged from 32 to 0.5 Hz spaced at 24 per octave. The feed-forward model had a preferred rate of 2 Hz.

Two complementary approaches were taken to this problem, the first an engineering control theory approach, and the second a neurobiological one. The essential concept was that beat induction and temporal tracking could be regarded as a form of sensory-guided action involving all those areas of the brain that are involved in planning movement (Todd et al., 1998, 1999, 2002). At the time of development in the mid to late 1990s there was not much auditory imaging or neuropsychological work to go on. There was, however, substantial work on visually guided action by John Stein and others (Miall et al., 1993; Stein, 1995). Figures 4, 5 are adapted from Todd et al. (2002) and illustrate the computation scheme and proposed instantiation in the brain. Figure 6 shows an output of the model to a fugue subject by JS Bach. Controversially at the time we argued that there must exist an auditory-motor pathway analogous to the visuo-motor projections involved in visually guided action, including the posterior parietal cortex, cerebellum, and premotor cortex.

### 1.4. Testing the theory

Following the development of the theory from 1994 to 2000, three strands of experimental work were conducted in parallel to test various aspects of it. The first experimental test was a comparison of the model with human performance. Todd et al. (2000b) reported an experiment in which a computational implementation of the theory was compared with the performance of two human subjects in a beat induction task for 160 samples of music. The model was quite successful but the study did reveal some weaknesses, including an inability to do scene analysis (Bregman, 1990). To avoid the confound of the scene analysis problem a second study was carried out where the model was compared with the performance of 20 human subjects tapping to samples of the 48 fugue subjects from the Well-Tempered Clavier (Todd and Lee, 2002). Overall the humans showed a wide distribution of performance compared with which the model was middle ranking.

In the second strand we looked for evidence that there was a relationship between how humans hear a rhythm and their bodies. A central prediction of the theory was that beat induction is mediated by the motion of an internal representation of the musculoskeletal system. Thus, in a population of individuals, there should be a systematic relationship between the size of the body and beat induction. An experiment was conducted by Todd and Cousins, (Unpublished manuscript) in the late 1990s to test this idea, later published in Todd et al. (2007b), which indicated that there was indeed a correlation between certain anthropometric factors and preferred tempo as measured in a purely perceptual task (see also Repp, 2007; Todd and Lee, 2007).

The third experimental approach to testing the sensory-motor theory was to look for evidence of movement related components in auditory evoked potentials. Todd and Seiss (2004) reported the results of an ERP experiment to investigate brain processes underlying beat induction. Subjects were required to listen to an anapest click rhythm under active and passive conditions, preceded by a condition in which the stimulus was unpredictable. A further condition was added which introduced uncertainty into the presence or absence of the third click. The results showed that the evoked auditory N2 potential was enhanced in regular as compared with random stimuli. Random omission of clicks on some trials suppressed the N2 but induced a later N3 prior to a P300 when the click was present. Comparing active and passive conditions indicated that pre-movement negativities (PMN) overlap spatiotemporally with the regular N2. They interpreted the potentials as being consistent with the operation of two distinct sensory-motor circuits: (1) an automatic, internally driven circuit involving supplementary motor area (SMA) for which the N2 may be a manifestation, and (2) an attention dependent, externally driven circuit involving posterior parietal cortex (PPC) for which the N3 may be a manifestation.

## 2. Subsequent developments

### 2.1. Beat induction modeling

#### 2.1.1. Linear filter-bank models

Following the original invention of the AM spectrometer as described in 1993/4 several later computational modelers attempted to improve or emulate these proposals. Smith and Kovesi (1996), Smith (2000), and Smith and Honing (2008) suggested use of the Morlet wavelet which he argued had better properties than the dual filter approach advocated. Although ideal from a mathematical point of view, such a wavelet could not, we believed, be a model of human perception as it is acausal. Cemgil et al. (2000) proposed an alternative wavelet approach, but which shared the acausality of Smith's approach. In a departure from the MIT autocorrelation tradition (Vercoe, 1984; Brown, 1993), Scheirer (1998) adopted several aspects of Todd's filter-bank approach, including the use of a damped sinusoid impulse response and the use of multiple frequency channels (Todd, 1996a). Unlike the wavelet approach though, Scheirer's (1998) filter-bank employed a comb filter and did not use a constant-Q, but rather a fixed damping half-time of about 1500 ms. As demonstrated by Todd and Brown (1996), however, the approximate Weber Law sensitivity to tempo in humans implies a constant-Q or wavelet representation. Many subsequent modelers who made use of a linear filter-bank approach (e.g., see Gouyon et al., 2006) inherited this design flaw in Scheirer's filter-bank. The autocorrelation approach (e.g., Goto and Muraoka, 1998), which is the Fourier sister of the power spectrum, also shares this flaw. Without any corresponding correction, such models are bound to be oversensitive to higher metrical harmonics and make many octave errors. Scheirer got round the problem by constraining the filter bank from 1 to 3 Hz, but as a result his system is unable to handle very fast or slow rhythms. Despite these flaws, however, of all the different modeling approaches considered, the linear filter-bank approach appears to be the most successful from an engineering perspective (Klapuri et al., 2006), and can therefore be considered a validation of the original linear filter concept, although a wider test of other approaches, including non-linear oscillator models has not been conducted (see next section).

Although the linear filter-bank approach has been proven in the various implementations that have appeared, these implementations all suffer from another important deficiency, which is that they have no motor representation. At least in part this is because the above were purely engineering approaches which were not based on a computational/neurobiological theory of human beat induction. An important distinction is one between an algorithm which is designed purely on engineering considerations, i.e., whatever works best, and an algorithm which is designed to implement a theory of how a biological system works, i.e., a computational model to test a theory. It was for this reason that the series of algorithms proposed by Todd and colleagues from the earliest included a component which could account for the existence region of the beat. This sensory-motor approach to beat induction also finesses the need to restrict the filter-bank to a limited range, as in the Scheirer (1998) and Klapuri et al. (2006) algorithms.

#### 2.1.2. Linear filter vs. non-linear oscillator models

It has been suggested that linear filter models are essentially a kind of oscillator model (Large, 2008), akin to the adaptive non-linear oscillator models originally proposed by Large and his colleagues and by McAuley (Large and Kolen, 1994; McAuley, 1995; Large and Jones, 1999). The Large and Kolen (1994) oscillator approach emerged from the broader dynamical systems theory (DST) developed to explain rhythmic movement coordination (Turvey, 1990; Kelso, 1995; Sternad, 2000). Proponents of DST argue that a motor component is implicit in their descriptions, which as we argued above is necessary for beat induction according to the sensory-motor theory. However, as pointed out by Large and Kolen (1994) the simple phase tracking of DST used to explain rhythmic movement coordination is insufficient to model beat induction. In addition, we believe, it is beyond the scope of DST to account for passive beat induction in the absence of explicit movement. Non-linear oscillator models of beat induction are often characterized as “neurodynamic” accounts, because they claim to described an internal brain process, rather than provide a description of external movement patterns typical of the DST paradigm, although they do share many mathematical features. Such models have become the mainstream explanation of the origin of beat induction and many variants have subsequently been developed (for review see Repp, 2005; Large, 2008; Repp and Su, 2013).

The argument that linear “resonators” are just a subset of oscillator models has some weight when the filters are high Q, as in the Scheirer (1998) and Klapuri et al. (2006) algorithms, or in the case of Langner and Goebl's (2003) “oscillating system.” The constant-Q or wavelet filter-bank, as in the Todd (e.g., Todd et al., 2002) or Smith (e.g., Smith and Honing, 2008) algorithms are, however, fundamentally different because the impulse response of the individual filter is more localized in time and has a scaling property. The collective output of a bank of such filters constitutes a passive wavelet transform of the original signal. Such impulse response properties can be seen in receptive fields within sensory cortex, as described above, and are thus plausible models of the representation of rhythmic patterns in the sensory (including auditory) areas of the brain.

It has also been suggested that non-linear oscillator models are superior to the linear filter approach because some syncopated rhythms do not have a spectral representation at the tactus level (Velasco and Large, 2011), with the result that in such cases linear filter models, unlike non-linear oscillator models, are unable to find the beat. In fact most rhythms do have at least weak spectral representation of the tactus, especially if a rhythm is repeated, thereby generating harmonics of the repetition frequency. A constant-Q filter system will be sensitive to the low repetition frequency and if combined with harmonic series pattern matching a missing harmonic could be replaced, especially if close to the preferred motor tempo. However, in cases where the tactus is indeed spectrally absent and linear models are therefore unable to find the beat, it is quite possible that human listeners would also have problems (see e.g., Fitch and Rosenfeld, 2007). A critical test for a model of beat induction is that it should fail where humans fail, otherwise it is just another beat finding algorithm rather than a psychologically plausible computational model. Non-linear models may well out-perform linear models with such rhythms, as Velasco and Large (2011) claim, but if they also out-perform humans, then they fail the test of psychological plausibility.

In more recent incarnations non-linear oscillator models have moved away from the original concepts of adaptive oscillators and “attentional energy” and focused on a growing literature based on EEG/MEG beta/gamma band studies (Snyder and Large, 2005; Zanto et al., 2006; Fujioka et al., 2009, 2012; Iversen et al., 2009). Such processes are fundamentally different in the frequencies involved, i.e., about 10–40 Hz, which are much higher than those normally associated with beat induction. They also appear to play a fundamentally different role from the original adaptive oscillator concept, primarily in signaling cortico-cortical binding for “audio-motor coupling,” rather than represent a beat induction process directly, although it has been suggested that the gamma band activity reflects “anticipatory entrainment” (Fujioka et al., 2012). For these reasons we believe that although the beta/gamma focus is superficially attractive, in that it is an attempt to relate non-linear oscillator accounts of beat induction to measureable brain processes, its explanatory power has been much weakened by this move (for a lengthier discussion see Todd and Lee, 2015).

In contrast, as we noted above, the linear filter approach can be related directly to the physiological properties of sensory cortex receptive fields which have parameters much closer to the beat induction existence region. When such representations are coupled with a motor representation, as in Todd et al. (2002), and if the motor output is allowed to feedback to the sensory representation, either by allowing the system to externally hear or feel itself, or by allowing internal motor reafference or corollary discharge, then some properties may appear in the distributed system, which the non-linear modelers claim is necessary to model rhythm (Large, 2008). Herein lies the fundamental disagreement we have with the non-linear oscillator “neurodynamic” approach. Such models ultimately create a mind-set which looks inwards within the brain for autonomous cells or anatomical structures which have the property of a clock, e.g., cell networks which generate 40 Hz, or structures such as the basal ganglia, which is argued to be the seat of an “internal clock” (Matell and Meck, 2004). Although not all oscillator theorists would subscribe to such anatomical specificity the fact is that such concepts are commonly held within the wider community. In contrast the sensory-motor approach creates a mind-set which looks outwards to the interaction of the body with the environment and to the distributed representation of the body and its environment within the brain. As we noted above, although DST considers perception-action coupling, it is beyond its capacity to explain passive beat induction in the absence of movement because it has no concept of internal representations. We dedicate the remainder of this paper to expanding the case for the sensory-motor theory.

### 2.2. Motor interpretations of rhythm perception

#### 2.2.1. Rhythm and “embodied cognition”

In the last decade or so there has emerged in psychology a current of thought which has been labeled “embodied cognition” (Leman, 2008). The Todd et al. (2007b) study can be seen as another example from within this trend. Many experiments have now been conducted that have provided further weight to the notion of a link between musical beats and the body. To mention a few, Styns et al. (2007) show a close overlap of locomotor rates and musical beat rates. Toiviainen et al. (2010) in an analysis of spontaneous body movements to music found distinct eigenmodes, with both vertical and rotational torso motions, as well as arm motion, being manifest at different metrical levels. Dahl et al. (2014) provide further evidence of a link between preferred dance tempo and the dimensions of the body, consistent with a natural frequency or eigenmode explanation of preferred tempo. We may also mention the study of McAuley et al. (2006) who present evidence that children have shorter preferred beat periods than adults, though they do not offer any theoretical explanation for the phenomenon. From the sensory-motor perspective a natural explanation of this is that children have shorter preferred periods because they have smaller bodies.

#### 2.2.2. Rhythm perception evokes brain areas involved in motor timing

At the time when we first formulated a neurobiological hypothesis for the sensory-motor theory there were relatively few imaging studies of rhythm perception (see Todd et al., 2002 for review). However, it was already well-understood that in sensory-motor synchronization, self-cued regular movements involved the SMA in contrast with externally-cued movements which involved greater activity in the PMC and IPL (Jäncke et al., 2000; Jenkins et al., 2000). Subsequent imaging studies of sensory-motor synchronization and continuation tapping essentially substantiated this position (Cunnington et al., 2002; Lewis et al., 2004). In the early 2000s the first evidence emerged that rhythm perception evoked the same areas as involved in motor timing even when there is no explicit movement (Schubotz et al., 2000). Such evidence has generally been interpreted within the wider framework of time perception and estimation where the SMA/basal ganglia are considered to be the locus of an internal clock (Harrington et al., 1998; Meck and Benson, 2002). What was not clear from this work was whether the same mechanisms could be invoked for more complex non-isochronous rhythms in a musical context and what was the connectivity to the auditory cortex.

In the mid-2000s the concept of an auditory-motor interaction emerged in the imaging literature, as reviewed by Zatorre et al. (2007). In Zatorre et al.'s (2007) scheme various pathways of connectivity between A1 and PMC and SMA, as well as prefrontal cortex, were proposed. Several outstanding issues were highlighted, including interactions between the PMC and SMA systems, the role of proprioception in integrating with auditory and motor components, and possible links to emotion. Schubotz (2007) articulated this most explicitly in a proposal which is essentially a restatement of the Todd et al. (1999, 2002) theory, although integrated with the most recent anatomical and physiological data then available. At this time several studies of beat induction were conducted which confirmed a role for SMA and basal ganglia (Grahn and Brett, 2007, 2009; Grahn and Rowe, 2009). Several other studies also showed that rhythm activates multiple motor areas of the brain (Chen et al., 2008; Bengtsson et al., 2009).

#### 2.2.3. Two distinct motor networks are implicated in rhythm and time perception

Although an audio-motor consensus has now been established it is also clear that there are two distinct sub-systems involved, involving respectively the cerebellum and basal ganglia and associated structures, but these are described in different terms. Lewis and Miall (2003) refer to cognitively and automatically controlled systems. Teki et al. (2011) propose distinct systems for “duration-based” and “beat-based” auditory timing. Grahn and Rowe (2013) propose two distinct systems for beat detection and beat prediction. In their account the beat detection system makes use of a widespread network of areas includes STG, IPL, PMC, and cerebellum, while the beat prediction system involves the SMA and basal ganglia. The existence of two such sensory-motor circuits is entirely consistent with the proposal by Todd and Seiss (2004), as noted in Section 1.4, but a point which we develop in Section 3.2.

### 2.3. Vestibular influences on rhythm perception

#### 2.3.1. The vestibular hypothesis rediscovered

The hypothesis that the vestibular system is central to musical rhythm as proposed by Truslit (1938) and independently restated by Todd (1992a, 1993b) was considered to be controversial (Repp, 1993). At least it was the source of considerable controversy at some early Rhythm Perception workshops (e.g., see Todd, 1992b). Despite this skepticism, however, the concept was reintroduced again independently a decade later in a series of papers by Phillips-Silver and Trainor (2005, 2007, 2008) and by Trainor et al. (2009). In a series of experiments initially conducted with infants, evidence was found that bouncing along with auditory rhythms influenced the perception of the rhythmic structure (Phillips-Silver and Trainor, 2005). Similar experiments were conducted with adults, where it was shown that only active and not passive movements had a significant influence on perception (Phillips-Silver and Trainor, 2007). More critically it was demonstrated that head movement and therefore activation of the vestibular apparatus was necessary to observe the effect (Phillips-Silver and Trainor, 2007), and further that vestibular influence could be achieved directly by using galvanic vestibular stimulation (Trainor et al., 2009). However, the matter remains controversial, with some authors suggesting that the vestibular influence is not direct (Trainor, 2007; Riggle, 2009; Trainor and Unrau, 2009).

#### 2.3.2. The vestibular sensory-motor network revealed

There has in the last 20 years been a very considerable number of studies using caloric, galvanic, and acoustic stimulation methods to investigate the anatomy of the central vestibular system (Lopez and Blanke, 2011; Lopez et al., 2012). This is remarkable given that it was not so long ago that the question was being asked if there was a vestibular cortex at all (Guldin and Grüsser, 1998). We now know that the primate and human central vestibular system is constituted by a complex sensory-motor network involving widespread cortical and sub-cortical structures. We will in Section 3.1 show how this is highly correlated with the networks underlying rhythm perception, but give below a brief anatomical description as an introduction.

The principal sub-cortical structures, upstream of the vestibular nuclei are the thalamus, the basal ganglia and cerebellum. Vestibular projections to the thalamus are extensive and thought to mediate a least three ascending projections. One may be involved in vestibulo-somatosensory and auditory-motor function, a second may be involved in vestibule-striatal motor function, and a third, in vestibulo-visual and visuo-motor function. A fourth pathway, via the VA-VL complex includes projections to cerebellum, basal ganglia, motor, and premotor areas.

These projections collectively converge on receiving areas in the cortex which in primates are mostly located in temporo-parietal and posterior insula, somatosensory, posterior parietal, anterior insula and lateral, and medial frontal cortex. The brain areas can be crudely categorized as lateral and medial. Within the lateral group are a temporo-parieto-insular and retro-insular area, parietal areas, including distinct posterior parietal (PPC) and a somatosensory area and frontal areas, including distinct vestibular premotor and anterior insular areas. Within the medial group are a mid-temporal, i.e., hippocampal and parahippocampal zone, a parietal, i.e., precuneus, zone and a large vestibular cingulate zone. This network, as well as subserving the processing of vestibular sensory signals of translation and rotation, also mediates cognitive functions of self-motion perception, navigation in space and gravity, and awareness of the self and body in space (Lopez and Blanke, 2011; Lopez et al., 2012).

#### 2.3.3. The vestibular system is responsive to sound and vibration

In the last 20 years since the vestibular hypothesis was revived in the early 1990s there has been what can only be described as a revolution in vestibular research. It was known in Truslit's day that the vestibular system could respond to sound, e.g., in the work of Tullio (1929), but also by others including Tait who proposed that not all hearing is cochlear (Tait, 1932). In the 1960s and 1970s vestibular acoustic sensitivity again came to the fore in auditory research when the so called inion response was found to be vestibular dependent and myogenic in origin (Bickford et al., 1964; Townsend and Cody, 1971). This work was given prominence by discovery of a closely related response referred to as a vestibular evoked myogenic potential (VEMP) (Colebatch et al., 1994). It was established that the VEMP was a manifestation of the vestibular-collic reflex mediated by acoustic sensitivity of the otolith organs and the vestibular spinal tract. For the following 10 years the VEMP was developed as a non-invasive clinical tool for diagnosis of, among other disorders, superior canal dehiscence (SCD). As well as being a clinical tool, the VEMP could also be used as a scientific tool to investigate the acoustic sensitivity of the otolith organsto both air (AC) and bone conducted (BC) sound. In a series of papers, Todd and colleagues showed that VEMPs from AC were tuned with a best frequency of about 500 Hz, that they could be activated by sounds found in the environment, and that there was a hedonic response to acoustic vestibular sensations (Todd and Cody, 2000; Todd et al., 2000a; Todd, 2001).

In an effort to look for cortical vestibular effects, new short-latency vestibular evoked potentials (VsEPs) were described (Todd et al., 2003; Rosengren and Colebatch, 2006). Further investigations showed that a major contribution to this was another myogenic response, but this time a manifestation of the vestibular ocular reflex (Rosengren et al., 2005, 2010; Rosengren and Kingma, 2013). From this emerged the ocular VEMP or OVEMP (Todd et al., 2004, 2007a; Todd, 2010, 2013). The OVEMP has since generated a huge number of publications and has been established alongside the old VEMP as an important clinical tool (Rosengren and Kingma, 2013). It remains controversial though, for a number of reasons, (see Todd, 2014). However, in a series of tuning studies Todd and colleagues found that in addition to the 500 Hz sensitivity to AC sound, there appeared to be another lower frequency sensitivity, especially to vibration at about 100 Hz (Todd et al., 2008b, 2009). Under the right conditions it could be shown that the system was so sensitive to 100 Hz vibration that responses could be obtained *below* the threshold of hearing for BC sound.

In parallel with the above work, efforts to look for VsEPs of cortical origin continued. Todd et al. (2008a) described short latency VsEPs produced by AC and BC sound. A source analysis indicated that these were dominated by the vestibular ocular reflex (VOR), and the central brain structures which were involved in VOR control, including the brain-stem/cerebellum. Most recently this work was extended to longer latencies with AC sound where it has been shown that vestibular receptors contribute to long latency auditory evoked potentials (Todd et al., 2014a,b). A source analysis confirmed that the short-latency responses were dominated by ocular/cerebellar effects but also indicated a large cingulate cortex source and a contribution from the STG. The presence of vestibular projections to the temporal lobe confirmed that the auditory and vestibular pathways are much more entwined than hitherto suspected, an observation which is relevant to vestibular influences on auditory rhythm perception (Trainor et al., 2009).

## 3. A new synthesis

In the preceding sections we have reviewed the history of the sensory-motor theory of beat induction and subsequent developments in the fields associated with rhythm and vestibular research. One thing is clear: 20 years on from its first formulation in modern guise in the early 1990s, the evidence is overwhelming that rhythm and beat perception is a sensory-motor phenomenon. Even when a listener is not overtly moving, the motor areas of the brain are co-active with sensory areas. Thus, the basic hypothesis is vindicated. There are, however, a number of outstanding issues, not least those highlighted by Zatorre et al. (2007). What is the role of proprioception and kinesthesis in integrating with auditory and motor components? How do the SMA and PMC systems interact? How does the sensory-motor activity link to motivation and emotion? In the following sections we seek to answer these questions in a new synthesis which brings together the various disparate strands of research.

### 3.1. Rhythm perception is vestibular

The starting point for the new synthesis is the observation that although the link between motor timing and rhythm perception has now been firmly established, the concept of rhythm perception is in most cases related to abstract mechanisms of timing, either of the clock type or oscillator type (e.g., Harrington et al., 1998; Lewis and Miall, 2003; Teki et al., 2011). Their claim is that components of the motor system have a more general cognitive timing function than just movement planning and execution. While this may be true in many contexts, for music from the sensory-motor theory perspective such timing or oscillation theories are almost entirely disembodied. We believe that such perspectives are incorrect and misleading for musical rhythm and that it is necessary to restore the Aristoxenian/Truslitian concept of self-motion to the center stage for the field to make progress, i.e., to put the body in the brain back into rhythm perception research. Schubotz's (2007) theory comes the closest as it has an explicit role for the body; however, the self and self-motion is not represented in this scheme.

In order to make this restoration we first show how the cortical and subcortical components of the rhythm network correlate very closely to the cortical and subcortical components of the vestibular sensory-motor network which mediates cognitive representations of the self in space. In what follows we consider each of 10 zones which are commonly shown to be activated in rhythm and vestibular studies.

#### 3.1.1. Superior temporal gyrus (STG)—auditory/rhythmic motion perception

The STG includes primary and secondary auditory cortex, which subsumes core, belt and parabelt areas (Rauschecker and Tian, 2000). That the vestibular system has inputs to STG is now also beyond dispute. Imaging studies, particularly using vestibular caloric and acoustic stimuli, consistently activate STG, including primary auditory cortex BA41 (e.g., Bottini et al., 1994; Suzuki et al., 2001; Fasold et al., 2002; Schlindwein et al., 2008). Both caloric and galvanic vestibular stimuli also active wider areas within STG, including BA 42, 22 (Lobel et al., 1998; Bense et al., 2001). Recent work by Todd et al. (2014a,b) confirmed with acoustic stimuli that there is a likely vestibular contribution to AEPs when above vestibular threshold. Thus, both anatomical and physiological studies confirm a convergence of cochlear and vestibular projections to STG. Within the rhythm literature STG is ubiquitous, as one would expect since most stimuli use sound inputs, but does auditory cortex play an active role in rhythm perception, and not just a passive reception role? When beat detection and prediction are dissociated the STG is more activated during detection (Grahn and Rowe, 2013). Indirect recording from the auditory cortex via EEG also indicates an active role (Nozaradan et al., 2011). Thus, given that the vestibular system has direct access to primary and secondary auditory cortex it is entirely plausible that vestibular inputs, either from sound or movement, could interact during rhythm perception or mediate a sense of auditory motion (Trainor et al., 2009).

#### 3.1.2. Hippocampus (HC)—memory for rhythmic motion

In addition to STG it has long been suggested that the mesial temporal lobe, and in particular the hippocampus, may play a role in the retention of rhythmic sequences (Penhune et al., 1999). The hippocampus has also been proposed as an important component in timing mechanisms (Yin and Troger, 2011). In animal studies vestibular inputs to hippocampus are well established physiologically (Cuthbert et al., 2000; Horii et al., 2004). In humans hippocampal responses to caloric and galvanic vestibular stimuli are commonly demonstrated (Vitte et al., 1996; Suzuki et al., 2001), and loss of vestibular input has been associated with loss of spatial memory (Brandt et al., 2005). Thus, auditory vestibular interactions within the hippocampus could plausibly have a role in the memory and retention of rhythmic/motional sequential patterns.

#### 3.1.3. The parietal insular vestibular cortex (PIVC)—sensory-motor transformations

In animal models the PIVC has been considered a core hub in the vestibular cortical network (Guldin and Grüsser, 1998). Much of the effort in human neuroimaging studies has been directed to the search for the human equivalent of primate PIVC (Lopez et al., 2012). Within these studies there is general agreement that it lies within the posterior insula and temporo-parietal junction, but there is considerable variability between studies in the exact location, covering superior temporal gyrus (STG), posterior insula, and inferior parietal lobule (IPL), including both areas BA 39 and 40 (Lopez and Blanke, 2011). Nevertheless, within auditory imaging studies it is now agreed that there are projections through the temporo-parietal junction or Tpt which play an important role in sensory-motor transformations as part of a “dorsal stream” (Hickok and Pöppel, 2007; Isenberg et al., 2011). These studies are entirely consistent with the prior proposals of Todd et al. (1998, 1999, 2002) which anticipated these and other similar imaging results (e.g., Zatorre et al., 2002; Scott and Johnsrude, 2003). Thus, the general coincidence between the vestibular PIVC and auditory Tpt areas is indicative that vestibular and auditory inputs share this core hub in the sensory-motor cortical network with its links to the frontal lobes and role in sensory-motor transformations. In rhythm imaging studies IPL is implicated, especially for non-metrical rhythms (e.g., Grahn and Rowe, 2013), which require attentional effort.

#### 3.1.4. Somatosensory cortex (SI)—proprioceptive self-motion perception

The vestibular somatosensory cortex is well-established in animal and human models. In the primate brain areas 2v and 3av are implicated approximately in the head and neck region of the homunculus. There is some variation though, with the hand and arm area and trunk areas being implicated in some species (Lopez and Blanke, 2011). In human imaging studies this area has been demonstrated using both caloric and galvanic stimulation (Bottini et al., 1994; Lobel et al., 1998), although not using acoustical stimulation. It is thought to represent the human equivalent of neck and hand, although this has not yet been clarified. For passive listening to rhythms primary somatosensory cortex is rarely implicated but if a subject is engaged in an active synchronization task there will be proprioceptive feedback. This can be seen in both imaging studies of cued and uncued movement and in evoked potential studies of sensory-motor synchronization (Jenkins et al., 2000; Praamstra et al., 2003; Todd and Seiss, 2004).

#### 3.1.5. Posterior parietal cortex (PPC)—exteroceptive self-motion perception

In the animal models area 7, VIP and MIP constitute the posterior parietal vestibular zone (Guldin and Grüsser, 1998). It is thought that the human homolog of primate area 7 is the angular and supramarginal gyri areas 39 and 40, constituting the IPL. Numerous imaging studies using caloric, galvanic, and acoustic stimuli have shown activation in the IPL (Grahn and Rowe, 2013). Such activations extend to human area BA 7, the superior parietal lobule, and medially to the precuneus. The precuneus has been identified with mental imagery, self-awareness and self-agency (Cavanna and Trimble, 2006). It has strong subcortical connections to the thalamus and basal ganglia, consistent with it being involved in self-action. It has also been associated with whole-body and self-motion perception (Jahn et al., 2004; Kovács et al., 2008), and electrical stimulation of this area can induce sensations of self-motion (Kahane et al., 2003). Although within the imaging studies primarily caloric stimulation has implicated precuneus, evoked potential studies using acoustic activation of the vestibular apparatus have indicated a contribution from this area (McNerney et al., 2011). Of particular interest is the fact that precuneus is active in both beat based and non-beat based rhythms (Grahn and Rowe, 2013). Thus, both auditory and vestibular signals may converge within precuneus to produce percepts of self-motion for both highly metrical rhythms and for non-metrical expressive music.

#### 3.1.6. Anterior insula (AI)—interoceptive self-motion perception

There is no animal homolog of the anterior insular area but it is consistently activated in human imaging studies by caloric, galvanic, and acoustic stimuli (Bucher et al., 1998; Bense et al., 2001; Suzuki et al., 2001). It is believed that anterior insula is principally associated with bodily awareness where visceral, proprioceptive, kinesthetic, and equilibrioceptive signals are integrated (Craig, 2009). It has been suggested that vestibular integration in this area contributes to the interoceptive perception of the self (Lopez and Blanke, 2011). Anterior insula has also been implicated in imaging studies of rhythm perception, particularly for non-regular unpredictable rhythms (Grahn and Rowe, 2013), and may be part of a wider region, including inferior frontal gyrus, which forms a ventrolateral corticolimbic process for switching between internally and externally oriented control (Tops and Boksem, 2011).

#### 3.1.7. Premotor cortex (PMC)—externally evoked rhythmic movement control

In monkeys, vestibular premotor areas include ventral premotor cortex (area 6v) and the frontal eye fields (FEF) and are thought to be associated with cortical control of the VOR and smooth pursuit, or reflex suppression during visually guided smooth pursuit. In human vestibular imaging studies premotor cortex (PMC) may be activated by caloric, galvanic and acoustic stimulation (Bense et al., 2001; Fasold et al., 2002; Miyamoto et al., 2007). Again it has been suggested that these areas may be involved in control of the vestibular reflexes. Within the auditory literature PMC is a major target of reciprocal connections, with the IPL as a component of the dorsal stream for externally guided action (Chen et al., 2006). This is a central component of the sensory-motor theory of rhythm perception as originally formulated by Todd et al. (1999, 2002) and in more recent guises, such as in Schubotz's (2007) motor prediction scheme. Thus, the vestibular system is well-placed to influence an auditory interpretation (e.g., as in Trainor et al., 2009), and PMC is active during both metrical and non-metrical rhythms (Grahn and Rowe, 2013).

#### 3.1.8. Cingulate and supplementary motor cortex (CMA/SMA)—voluntary and reward based rhythmic movement control

In the animal literature the cingulate cortex (which is part of the mesial frontal cortex) is considered to be a core vestibular area because it is densely connected to PIVC, and also to somatosensory and visual parietal areas (Guldin and Grüsser, 1998). It is believed within the animal models that there is no direct thalamic input and that its activity is primarily the result of the cortical connectivity (Lopez and Blanke, 2011). In human imaging studies both anterior (Bottini et al., 1994; Bense et al., 2001; Emri et al., 2003; Miyamoto et al., 2007) and middle cingulate areas (Suzuki et al., 2001; Fasold et al., 2002; Indovina et al., 2005; Stephan et al., 2005; Miyamoto et al., 2007) are implicated using caloric, galvanic, and acoustical vestibular stimuli. It has been suggested that the role of the cingulate cortex in vestibular processing is for the integration of visual perceptual and self-motion cues (Lopez and Blanke, 2011). The cingulate and the supplementary motor areas (SMA) have long been recognized as having a major role in self-willed motion (e.g., Passingham, 1993; Jenkins et al., 2000). SMA and cingulate motor area (CMA) (which is a part of the cingulate cortex) is also confirmed as being the locus of the PMN including the “Bereitschaftspotential (Kornhuber and Deecke, 1990).” Within the rhythm literature, both cingulate and SMA are strongly implicated particularly for beat based rhythms (Grahn and Rowe, 2013). Todd and Seiss (2004) in their source analysis of the N2 associated with beat induction suggested both cingulate and SMA sources. Most recently Todd et al. (2014a,b) indicate a cingulate source contributing to AEPs when above vestibular threshold.

#### 3.1.9. Cerebellum (CB)—feedforward models of rhythmic movement

The CB is a major recipient of vestibular afference (Büttner-Ennever, 1999). This connectivity allows the vestibular system to have a major role in the control of eye movement, particularly the gain of the VOR, and body posture (Büttner-Ennever, 1999). Human neuroimaging studies also strongly implicate CB, particularly within lobule VI of the anterior lobe, which is thought to play a role in sensory-motor, spatial, and emotional processing (Lopez et al., 2012). Source analysis of VsEPs from sound consistently implicates the cerebellar-brainstem complex as contributing to short latency responses associated with OVEMPs ((Todd et al., 2008a, 2014a),b). The CB has long been associated with having a cognitive timing function and in musical rhythm perception (Ivry et al., 1988; Penhune et al., 1998), as well as being central to sensory guided action (Miall et al., 1993; Stein, 1995). Imaging studies of musical rhythm consistently also implicate the cerebellum, especially when the rhythms require attentional processing (Keller and Burnham, 2005; Grahn and Rowe, 2013).

#### 3.1.10. Basal ganglia (BG)—repository for habitual rhythmic movements

In the animal literature the basal ganglia have not been considered as a classical vestibular zone. There are, however, a few studies which have demonstrated vestibular processing in the putamen (e.g., Liedgren and Schwarz, 1976). Anatomical studies indicating connectivity of the striatum to the insula have also implicated a possible vestibular link (Lopez et al., 2012). However, within the human imaging literature putamen activation is reported during self-motion perception during CVS and GVS (Bottini et al., 1994; Bense et al., 2001; Dieterich et al., 2003). Activity of the basal ganglia along with the SMA during self-cued movements has been reported in many studies (Jenkins et al., 2000), and the basal ganglia, like the cerebellum, has also been implicated in time perception (Ivry and Keele, 1989; Harrington et al., 1998). However, in a musical context the basal ganglia are strongly implicated in beat induction Grahn and Brett (2007), and more recently specifically with beat “feeling” rather than finding (Grahn and Rowe, 2013).

#### 3.1.11. Summary

To summarize then, in the above section we have shown that for each of the brain areas associated with rhythm perception there is a close correlation with the vestibular sensory-motor network. These correlations we have summarized in Table 1, which also shows the likely thalamic or sub-thalamic vestibular input route and putative function. A number of general principles emerge from this analysis. The first is that the vestibular apparatus has a privileged access to the entire network via multiple areas of the thalamus or more directly to the sub-cortical components. In contrast the cochlea has a much more limited access to the network, primarily via one station in the temporal lobe and subsequent cortico-cortical connections. Clearly therefore, vestibular inputs, either by acoustic, gravitational or inertial stimuli are strongly able to influence the rhythmic interpretation of an auditory input.

**Table 1:** **Summary of areas and putative role (internally vs. externally referenced), with routes for cochlear vs. vestibular input (thalamus)**.

**Region**	**Area**	**Vestibular zone**	**Vestibular thalamic relay**	**Rhythm/motional function**
Temporal	STG	Superior temporal cortex	MGB	Auditory rhythm/motion detection
	MTG	Hippocampus	AD, PH	Memory for self-motion in space
Temporal/parietal	Tpt/TPJ	Posterior insula and temporal-parietal cortex	MGB	Rhythmic/motional audio-motor transformation
Parietal	IPL	PIVC	VPL/Vim/IL	Rhythmic/motional audio-motor transformation
	SPL	Precuneus	VPL/pulvinar	Exteroception of rhythmic self-motion
	PCG	Somatosensory	VPM/VPL	Proprioception of rhythmic self-motion
Frontal	PMC	Premotor cortex	VA-VL, shared with CB	Externally evoked rhythmic movement control
	SMA/CMA	Cingulate area motor	VA-VL, shared with BG	Internally evoked rhythmic movement control
	Cingulate Cortex	Cingulate area limbic	IL (MD?)	Limbic responses to rhythmic movement
Frontal/temporal	IFG/aSTG	Anterior insula	VPI/VM	Interoception of rhythmic self-motion
Sub-cortical	Cerebellum (CB)	Floculus/nodulus vermis	Direct input + fastigial nucleus	Forward model of body for motion prediction
	Basal ganglia (BG)	Striatum	IL + direct input to NAc via PBN	Habitual rhythmic/motion responses

*AD, anterior dorsal nucleus; BG, basal ganglia; CB, cerebellum; CMA, cingulate motor area; IL, intralaminar nucleus; IFG, inferior frontal gyrus; IPL, inter-parietal lobule; MD, medial dorsal nucleus; MGB, medial geniculate body; MTG, medial temporal gyrus; NAc, nucleus accumbens; PBN, parabrachial nucleus; PIVC, parietal insular vestibular cortex; PH, posterior hypothalamic nucleus; SMA, supplementary motor area; SPL, superior parietal lobule; STG, superior temporal gyrus; TPJ, temporo-parietal junction; Tpt, temporo-parietal area; VA, ventral anterior nucleus; Vim, ventral intermediate nucleus; VL, ventral lateral nucleus; VM, ventral medial nucleus; VPI, ventral posterior inferior nucleus; VPL, ventral posterolateral nucleus; VPM, ventral posteromedial nucleus*.

A second principle which emerges is that areas within the rhythm/vestibular sensory-motor network can be divided into two subnets, one which is externally referenced, i.e., STG, hippocampus, IPL, cerebellum and PMC, and a second set which are internally referenced, i.e., precuneus, SMA/CMA, cingulate cortex, basal ganglia, and anterior insula. This subdivision is not strict, as some areas could face inwards and outwards, so to speak, but there is natural alliance between those areas which collectively represent the self in motion, e.g., precuneus, anterior insula, and cingulate cortex. This group of areas are also strongly linked to the limbic system and therefore likely mediates the link between self-motion and emotion (Koelsch, 2014).

### 3.2. Beat induction is mediated by externally vs. internally guided motion of the body in the brain

The final point in the summary above leads naturally to the second proposition in the new synthesis, i.e., that beat induction is mediated by two distinct sensory-motor circuits, and as was noted also in Section 2.2.3 the existence of two circuits is fully established in the literature. In the rest of this section we explore in detail the proposition that rhythm perception evokes two parallel sensory-motor systems which mediate the *external* vs. *internal* guidance of motion of the body. We do so by collating the evidence that both circuits can be described anatomically as being composed of multiple somatotopic representations.

**Figure 7:**
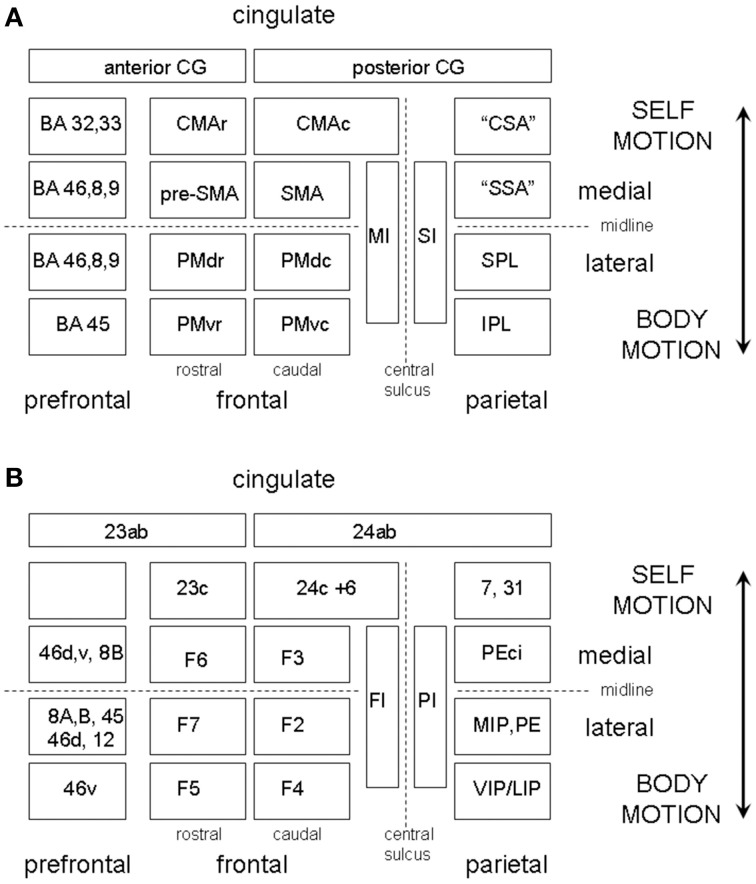
**(A)** A highly schematic representation of somatotopically organized sensory and motor cortical areas organized on a medial vs. lateral and frontal vs. parietal oriented map of one hemisphere. The map is bounded medially by the cingulate gyrus and frontally by the prefrontal cortex. The frontal areas are further partitioned according to their rostral or caudal location in the map. The PMC is divided into four regions, labeled PMdc, PMdr, PMvc, and PMvr. The SMA/CMA region is similarly partitioned into CMAc, CMAr, pre-SMA, and SMA. The caudal motor areas each have a corresponding parietal receiving area, which we have labeled the “cingulate sensory area” (CSA), the “supplementary sensory area” (SSA), the SPL, and IPL. **(B)** Shows the same as **(A)** but with the equivalent non-human primate labels attached.

The overall scheme that we envisage is illustrated in Figure 7 which shows in simplified form the somatotopic sensory and motor cortical areas organized on a medial vs. lateral and frontal vs. parietal oriented plan of one hemisphere. The lateral motor maps are the PMC areas and the medial the SMA/CMA areas. The frontal areas are further partitioned according to their rostral or caudal location in the map and each of the four caudal PMC or SMA/CMA body maps is paired up with a parietal body map. Each of these participate in parallel sensory-motor circuits, but their lateral or medial anatomical location in the hemisphere corresponds physiologically to their external or internal reference, i.e., to externally triggered or voluntary internally motivated movement.

The two beat induction circuits we propose are instantiated in respectively auditory-parieto-cerebellar-premotor pathways (beat finders) vs. auditory-striatal-cingulate-motor pathways (beat keepers or “internal dancers”). The two systems are linked at various cortical and subcortical levels so that when a regular beat is detected it is rapidly transferred from the external to internal system. Both internal and external guidance pathways can be synchronized by direct vestibular input, either by acoustic (sound/vibration) or inertial (body movement) activation of vestibular receptors. We consider each in turn.

#### 3.2.1. The beat “finders,” external guidance of the body in the brain

The beat finder circuit is essentially that which was described in Todd et al. (1998, 1999, 2002). As described above, a “dorsal” stream projects through the Tpt/IPL region during sensory-motor integration. This region already has well-defined somatotopy which parallels that for SI (Eickhoff et al., 2008). This somatotopy extends posteriorly in the parietal cortex. In response to moving body parts somatotopic organization is preserved in SII, with activation also in inferior and superior parietal lobules (Ruben et al., 2001). Thus, even without consideration of motor representations, an auditory/vestibular rhythm may evoke an internal representation of the body in motion. However, given the strong connectivity between PPC, the cerebellum and frontal cortex any such posterior parietal representations are linked to corresponding somatotopically organized motor representations (Ruben et al., 2001; Wheaton et al., 2004).

As for the somatosensory homunculus, the motor homunculus in MI is well-organized, and this organization is repeated anteriorly in pre-motor cortex (Muakkassa and Strick, 1979). As illustrated in Figure 7 the PMC is divided into four regions, labeled F2 (or PMdc) and F7 (or PMdr) in dorsal PMC, and F4 (or PMvc) and F5 (or PMvr) in ventral PMC. PMvc and PMvr were discovered to contain so called “mirror neurons” (Rizzolatti et al., 1998, 2002). Each of these areas has a somatotopic organization and a corresponding parietal receiving area but with F7 being more specific for eye movement and being more prefrontally connected (Rizzolatti and Luppino, 2001). F4 (PMvc) is part of a parieto-frontal circuit involving body part specific goal directed actions. In humans the PMvc equivalent also shows a well-defined somatotopy, where head movements are represented ventrally and arm movements dorsally (Bremmer et al., 2001). PMvr in humans is thought to correspond to the speech area BA 44, but also responds to hand movements in primates. The parietal receiving areas for F2 (PMdc) likely corresponds in humans to BA 5 and 7 in SPL. Given that the SPL is more associated with an internal sense of the body in space, it may be that an important distinction between the PMdc and PMvc systems is that the PMdc is more associated with whole-body motion through space while the PMvc represents the motion of the body parts to the center. There are thus potentially four parallel externally oriented somatotopically organized sensory-motor circuits which may be engaged in rhythm perception, mediating whole body, eye, body part, and speech or singing gestures.

The final component in the externally referenced system is the cerebellum, for which somatotopy is also very well-established (Manni and Petrosini, 2004). The cerebellum, as described above, has the role of feedforward model in the external guided sensory-motor circuits. There appear to be two maps of the body, one located in the anterior and one posterior cerebellum. The anterior homunculus appears to be most related to motor/premotor activity, but both cerebellar homunculi receive vestibular inputs.

#### 3.2.2. The beat “feelers,” internal guidance of the self in the brain

The starting point for this component of the theory is the hypothesis which emerged from the Todd and Seiss (2004) experiment, i.e., that the N2 represents a readiness for action cognitive reflex which may become entrained to form a pre-movement negativity. The generators for the N2 were located to ACC and SMA. Näätänen and Gaillard (1983) first suggested that the N2 was an orienting reflex, but new analyses by Todd and Lee (2015) provide strong evidence that the N2 includes a vestibular dependent component that is generated in cingulate cortex. For regular rhythms this reflexive readiness activation in the cingulate facilitates the rapid transfer from the external guidance pathways in PMC and develops into an entrained premotion activity in CMA/SMA. Unlike the PMC systems, the CMA/SMA systems strongly feature the basal ganglia as a sub-cortical component.

In the non-human primate, F3, the homologue to SMA, has its parietal receiving area in PEci in the posterior cingulate sulcus (Rizzolatti and Luppino, 2001), which probably corresponds to the human dorsal posterior cingulate and precuneus (Parvizi et al., 2006). PEci has been referred to as a “supplementary sensory area” (SSA). Both F3 and PEci are somatotopically organized in the primate (Rizzolatti and Luppino, 2001), and both SMA and precuneus are strongly connected to the basal ganglia. Given the role of the precuneus and posterior cingulate in navigation in space and self-awareness, a SMA-SSA sensory-motor circuit based on these areas can mediate internally willed self-engagement of the body. SMA and precuneus are both established as having vestibular inputs so that activation of this circuit will also be associated with a sense of self-motion.

In addition to the SMA body map there is a pre-SMA region and two distinct CMA regions, CMAr and CMAc each of which is somatotopically organized (He et al., 1995; Hatanaka et al., 2003; Arienzo et al., 2006) (see Figure 7). CMAc has reciprocal connections with precuneus (area 7m) and area 31 (Parvizi et al., 2006), which by analogy we refer to here as the “cingulate sensory area.” This area is implicated in ego motion and can be activated by optic flow. Thus, CMAc can participate in a closed sensory-motor loop system in parallel with the SMA. An association with both SMA and M1 suggests that its function is associated with the execution of voluntary self-movements. In contrast CMAr, in common with pre-SMA, is relatively weakly connected to parietal areas and more strongly connected to prefrontal areas (Takada et al., 2004). It also, however, has a close proximity to the anterior cingulate gyrus. For this reason it is believed that CMAr plays a role in reward based selection of voluntary actions (Hatanaka et al., 2003). The CMAr is therefore a vehicle for vestibular reward to influence motion selection.

In addition to the somatotopy of the motor cortices, each of the four nuclei of the basal ganglia, i.e., the striatum, the pallidum, the subthlamic nucleus (STN) and the substantia nigra (SN), are also somatotopically organized (Nambu, 2011). It has been shown that the putamen, which is the input station for the motor loop, has at least two complete body maps corresponding to the M1 and SMA homunculi. In addition pre-SMA, CMAc, and CMAr have significant representations. These maps intersect in the striatum in a manner which reflects the functional division outlined above, so that the CMAc body map overlaps with M1 and SMA, while the CMAr maps in with pre-SMA. Of particular relevance to the sensory-motor theory of rhythm is the observation that PMdc (whole body) and PMvc (body part) areas also map into the SMA representation. This therefore provides a locus for rapid transfer of information within the putamen from the externally guided body-motion “beat finder” system to the internal guided self-motion “beat feeler” system. As noted above, vestibular activation of the putamen has been demonstrated for CVS and GVS and is also activated during optokinetic vection (Brandt et al., 1998; Kovács et al., 2008; Lopez et al., 2012). Thus, activation of the putamen is clearly associated with sensations or illusions of self-motion.

The STN also contains two complete body maps corresponding to MI and the SMA. As for the putamen, CMAc projects in with the M1 and SMA maps (Nambu, 2011). Within the motor region of the globus pallidus both GPe and GPi contain distinct and complete body maps for MI and the SMA. The head region in all cases is located ventrally but the GPi head region is continued into the SNr, which is contiguous with it anatomically. Thus, the SNr contains primarily a map of the head from the motor loop and it is established that active or passive movements of the head produce responses in GPi and SNr (Nambu, 2011). The SNc only does not appear to have a somatotopic organization. The SNr/GPi returns influence to the cortex via the VL-VA nuclear complex. The VL thalamus shows at least two well-defined body maps which appear to be segregated according to basal ganglia or cerebellum inputs (Asanuma et al., 1983). As described above the VL-VA complex is one of the major ascending thalamic projections of the vestibular system.

From the above then, we can see that at each stage of the motor basal ganglia loop there is both a strong topographic body map organization and a high degree of correlated vestibular input, which appears to be primarily targeted at the head regions within the maps and has its strongest influence at the input, i.e., striatal, and output, i.e., thalamic, stages of the loop. From a functional point of view the basal ganglia is a learning mechanism and repository for habitual, stereotyped goal directed motions selected by the SMA/CMA. As a component within the internal guidance sensory-motor circuit for self-action the stereotyped self-motion motions are likely to be simple whole body motions which involve the head in particular. Such habitual self-motions might at their simplest just be a kind of head bobbing, which is ubiquitous in human responses to music which has a beat.

### 3.3. The limbic connection—the “dance habit”

In the above section we described a theory of how beat induction is mediated by the activation of habitual motions of somatotopic body maps. Once these become active there is a very strong, almost reflexive compulsion to actually move, i.e., for the primary M1-S1 circuit to become active. As soon as the head is actually moved, or the vestibular system activated acoustically, vestibular inputs provide an additional reinforcement signal. This last statement provides a clue to the motivation and drive of beat induction since the CMAr is involved in the voluntary movement selection based on reward. Voluntary head bobbing, and other whole body motions involving the head are intrinsically rewarding and self-reinforcing because the vestibular system has inputs to the limbic system via both cortical and subcortical pathways. In the rest of this section we describe first the subcortical network. We then describe how the internal guidance sensory-motor pathway may be further linked to the limbic system by limbic and associative basal ganglionic circuits (Balaban and Yates, 2004; Yin and Knowlton, 2006).

**Figure 8:**
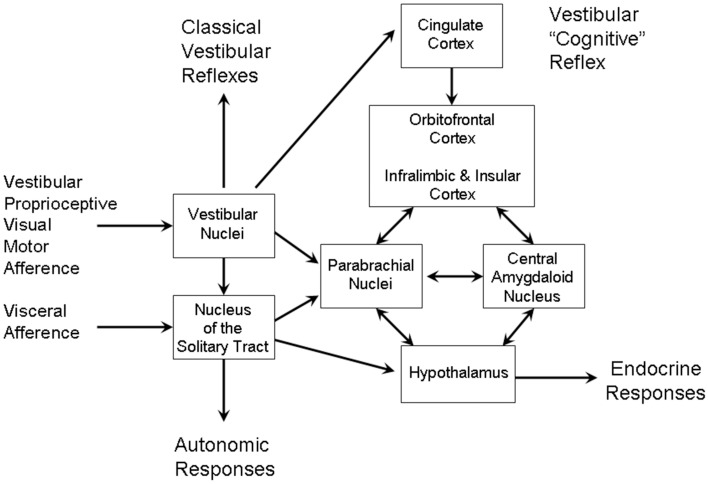
**The vestibular-parabrachial pathways that mediate the vestibular autonomic reflex**. Adapted from Figure 1 of Balaban (2002).

The vestibular-parabrachial network has been described in detail (Balaban, 2002) (see Figure 8). The vestibular nuclei, as well as projecting to the classical reflex pathways through the vestibular-ocular and vestibular-spinal pathways, also project sub-cortically to nucleus of the solitary tract (NTS) and the PBN. In these relay nuclei, vestibular inputs converge with visceral and gustatory afferents. These mediate vagal autonomic responses via the solitary tract and endocrine responses via NTS and PBN projections to the hypothalamus. The PBN may project directly to the NAcc, but also via the VTA. The PBN also projects to the amygdala and directly to infralimbic and insula cortex. As well as participating in the dopaminergic pathways, the vestibular nuclei also participates in serotonergic pathways via the raphe nuclei and noradrenergic pathways via the locus coeruleus (Balaban, 2002).

The limbic branch of the basal ganglion system has as its principal cortical area the orbital frontal cortex (Alexander et al., 1986). The loop is made up of the NAcc, the ventral STN, the ventral pallidum and mediodorsal nucleus (MD) of the thalamus which projects back to the OFC. In the same way that the vestibular system has a strong influence on the motor loop, so too with the limbic loop. The NAcc receives inputs from many other cortical areas, including the hippocampus, STG and orbitofrontal cortex, the first two of which have strong vestibular inputs. Vestibular inputs may also have an influence on the MD nucleus, although not as strong as the influence on the VA-VL complex.

Thus, we may see that collectively these multiple influences on limbic, autonomic, and endocrine systems by the vestibular system may mediate a number of affective responses to motion, both positive and negative, as well as normal vestibular sympathetic responses to postural change. Negative symptoms include motion sickness, vertigo and a number of anxiety disorders. Among the positive consequences are reward obtained from the viscero-vestibular activation from whole body motion. At low intensity, motion can be relaxing, such as in the case of cradles and rocking chairs. In more moderate intensities the effect is more stimulating, such as may be found at fun parks, bungie jumping etc. If however, the intensity of stimulation is large the effect becomes negative, especially if there is a visual-vestibular disparity as in sea sickness.

The fact that these effects have the appearance of responses to drugs, such as alcohol, should not be surprising given that the effects of addictive drugs are mediated by the same mechanisms as described above which mediate vestibular-pharmacological activations. The link between vestibular reward and addictive drugs is of course found also in the case of psychomotor or “dance” drugs such as ecstasy and amphetamine, which act by increasing the amount of synaptic dopamine available. Theories of the mechanisms of addition have traditionally been based around the concept of “pathological usurpation of neural processes that normally serve reward-related learning” or “maladaptive habit formation” involving dopaminergic circuits, specifically the NAcc and VTA, dorsal striatum and prefrontal cortex (Hyman et al., 2006). Pathological head bobbing or rocking, such as seen in some individuals with learning disorders, could be seen as a form of maladaptive vestibular self-stimulation. Dance drugs work in otherwise healthy humans because they amplify the normal effects of activation of the VTA/NAcc circuits during vestibular self-stimulation, e.g., dancing. We suggest, however, that vestibular self-stimulation and habit formation is a natural process, for which drugs are not necessary. Indeed, we believe that the vestibular self-stimulation or “dance habit” is learned at quite an early age and plays an important role in beat induction in human adults because it allows for a rapid reward based body self-motion selection in the SMA/CMA sensory-motor circuits.

**Figure 9:**
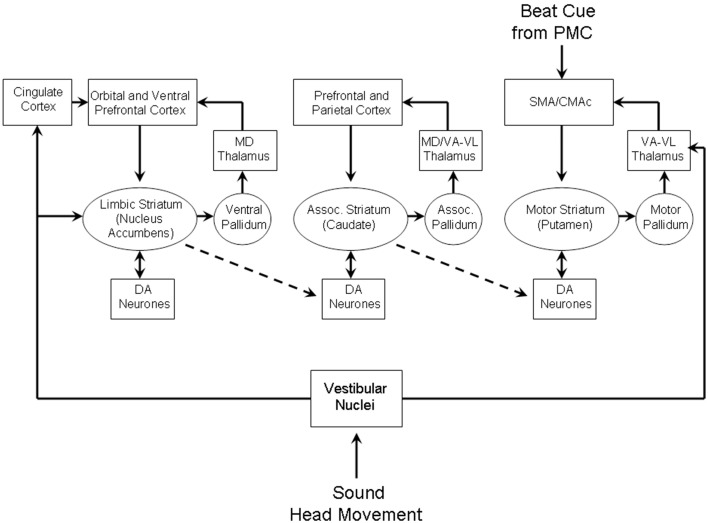
**Cortico-basal ganglia networks which may mediate vestibular habit formation**. Adapted from Figure 3 of Yin and Knowlton (2006).

More recent theories of habit formation include the idea that the parallel basal ganglia loops are not independent (Figure 9), but in fact have connectivity between them which allows for information to be transferred between them (Yin and Knowlton, 2006). The connectivity between the limbic to associative loop is via connections from the NAcc to dopaminergic neurons in the prefrontal zones in the SN. In turn the connectivity between the prefrontal loop to the sensory-motor loop is via connections from the caudate nucleus to motor areas in the SN. This allows a reward based learning mechanism to habituate a sensory-motor stimulus-response. We suggest that such a mechanism could form the basis of the dance habit. Essentially the proposal is that learning in the limbic loop based on vestibular reward, i.e., innate signals of positive affect from motion, is transferred to the sensory-motor loop as a learned beat cue stimulus to self-motion response via the associative prefrontal loop (see Figure 9). Once the dance habit is formed, when an appropriate beat cue is detected the requisite motor plans for self-motion are generated which will produce an anticipated reward on execution. Direct vestibular input from an actual movement (dance) also reinforces the “dance habit.” This mechanism thus explains the compulsion to move to a beat cue.

### 3.4. Vestibular reward is inherited—the origin of rhythmic behavior

In reviewing the case that brain areas associated with rhythm perception are highly correlated with the vestibular brain networks, that beat induction can be seen as a form of external and internally guided action in multiple sensory-motor circuits in which the vestibular inputs are represented in a privileged manner, and that a vestibular reward learning mechanism explains the compulsion to move to a regular beat, it is clear that the vestibular system has an absolutely central role in this scheme. To the reader not familiar with vestibular research this might seem astonishing or even incredible e.g., from a cognitivist perspective a direct link between the body and perception via the vestibular apparatus appears “naïve” (Honing, 2009). However, the clinching argument in this position is that when considered from an evolutionary perspective it is quite natural that the vestibular system should be central to rhythm. In this section we describe how the vestibular reward mechanism is inherited from anamniote (fish and amphibian) ancestors, for whom vestibularly mediated acoustic/movement behavior evolved through sexual selection.

The vestibular sense of vertebrates is one of the oldest senses. It certainly predates the mammalian cochlear system and almost certainly predates in evolutionary terms even the origin of vertebrates (Lewis et al., 1985). Practically all 36 phyla within the animal kingdom, perhaps apart from sponges, contain species that have a graviceptive organ, often in the form of a statocyst, known to be possessed by for example, by cnidarians, molluscs, arthropods, annelids, and holothurians, just to name a few. Any non-sessile animal organism must be able to orient in space for navigation, i.e., to know up from down, if it is to be successful in capturing prey or avoiding predation. This is obviously the case for visually based navigation, where a visuo-graviceptive interaction is required to accurately separate self-motion from motion of the environment, but the same is also true for an animal that cannot rely on light and which navigates by means of olfaction, such as some species of worm or parasitic animals, e.g., rhotists, where a chemo-graviceptive interaction takes place. However, in addition to exterocepto-graviceptive interactions, there must also be interocepto-graviceptive interactions so that appropriate control is maintained over the internal environment, depending on the animal's orientation in space (Balaban, 2002). Thus, even the most primitive nervous system must integrate the vestibular with visual, chemical and visceral sensory information. In more complex animals, such as chordata, vestibular senses must of necessity, therefore, be intimately linked to the autonomic and endocrine systems, as well as to the external senses.

It is this necessary vestibular-autonomic outcome of natural selection, we suggest, that provides for the vestibular system to then become a vehicle for sexual selection, because the appropriate vestibular-endocrine circuits are in place, and thus to be the origin of dance. Within all vertebrate classes examples can be found of reproductive behavioral displays which could in the most general sense of the word be described as dances. Invariably they involve body motions including the head, which therefore also are a form of vestibular self-stimulation. Invariably also such motions are accompanied by sound or vocal displays. One of course immediately thinks of bird mating dances, but examples can be found throughout the vertebrate phylum, not least from fish. This may seem remarkable, as not only do fish not have a cochlea, or any organ remotely homologous to a cochlea, but they also have no larynx, and yet many species are good hearers, and highly vocal, particularly for the purpose of reproduction. How can this be so?

To answer the first question on hearing without a cochlea we should note that the morphology and biophysics of graviceptors, which evolved independently more than once, are remarkably uniform. It simply requires within the organism a patch of tissue which is more dense than the surrounding tissue and some sensory receptors which can detect a positional change of the denser to less dense region when the animal changes orientation within the gravitational field or the animal accelerates (Fritzsch, 1999). In the case of fish this organ is referred to as an otolith organ and there are three, the saccule, utricle, and lagena. Each of these effectively is a kind of accelerometer. However, an accelerometer is also a sound sensor, since it must of necessity also respond to acoustical particle accelerations from the acoustic near-field (Horowitz et al., 2007) (By the Einstein equivalence principle a tilt and a linear acceleration cannot be distinguished). Mechanically it will have a resonance frequency which will be the frequency at which it is most sensitive to near-field particle motions. In many fish this is around 500 Hz. Thus, a graviceptor also becomes an audio sound sensor.

To detect the acoustic far-field (i.e., pressure field), though, a further adaptation is required, i.e., a device for transforming acoustic pressure to displacement (Fritzsch, 1999). A gas bubble is one such mechanism. In the case of fish the swim bladder serves this purpose, and is a good example of how anatomy can be adapted for an entirely different purpose. The swim bladder's effectiveness in communicating sound pressure to the otolith organs is enhanced in otophysines, of which the goldfish (*Carassius auratus auratus*) is an example, by means of a Weberian ossicle, a bony structure which couples the bladder directly to the inner ear (Popper and Fay, 1999). These adaptations allow such fish to hear remarkably well, and sufficiently for conspecific vocal communication to become biologically important.

To answer the second question on vocalizing without a larynx, fish can produce sounds in a variety of manners, but one of the most effective is to make use of the swim bladder as a drum. Thus, the swim bladder is both a receiver and a sender of sound. A large number of species make use of this method by means of a special sonomotor muscle, the cranial nerve supply of which is the same as the mammalian larynx, in order to produce drumming sounds. A good example of this comes from the haddock (*Melanogramus aeglefinus*). In this case it is only the males that vocalize but it is particularly important for reproductive vocal behavior since the drumming sounds can induce the female to release eggs, at the same time that the male releases sperm. As well as powerfully illustrating the biological importance of acoustic communication in fish, it demonstrates that an organ which was originally a graviceptor has through natural and sexual selection now become an organ through which ritualized or stereotyped sound and movement become a means for conspecifics to reproduce.

The acoustic mating behavior in fish is almost certainly mediated by a projection from the eighth cranial nerve (NXIII) to the hypothalamus, which controls the release of the appropriate hormones to control the release of eggs and sperm (Pitcher, 1986). This projection is likely mediated by the secondary gustatory nucleus (or secondary visceral nucleus) which is present in all species of bony fish (osteichthyes) and is likely the homolog of the parabrachial nucleus in amniotes (Voogd et al., 1988). It has been confirmed in teleost fish as having all the appropriate connectivity, including afferent signals from the NTS, and projections to the hypothalamus. Thus, we can already see in fish all the appropriate anatomical, physiological, and neurobiological apparatus to mediate reward from vestibular activation, but it is within amphibians that the comparative anatomy and homology of the PBN becomes fully clarified (ten Donkelaar, 1988a). Amphibian vocal reproductive behavior is, of course, commonly known and various proposals have been suggested for the pathway which mediate this, including the existence of what has been termed a secondary isthmal nucleus (Neary, 1988), also established to be equivalent to the PBN. Of particular importance is the fact that in amphibians the PBN projects to the N Acc and striatum (ten Donkelaar, 1988a), thus showing that the vestibular reward mechanism in amniotes (reptiles, birds, and mammals), including humans, is inherited from anamniote (fish and amphibian) ancestors (ten Donkelaar, 1988b; Voogd et al., 1988). It is beyond the scope of this article to provide details of the evolution of these pathways in amniotes but see Todd and Merker (2004) and Todd (2001, 2007) for further discussions.

It should also be noted that the existence of vocal mating behavior in fish is not limited to just male female pairs but also to shoals of fish. Fish chorusing by groups of males during the spawning season has been documented in many species, e.g., from the croakers and drummers (Sciaenidae) and grouper (Serranidae) families (Lociasso and Mann, 2011). The individual males can also adjust the calling rate depending on the competition strategy (Jordao et al., 2012). Many of the choruses also display periodicity indicating that the fish have become synchronized, consistent with the theory that this gives a reproductive advantage (Merker et al., 2009). The periodicity rates tend to be faster than in human synchronized vocal behavior, but this is consistent with the sensory-motor theory that individuals will synchronize with natural locomotor frequencies, in this case for the fish swim cycle. Fish also have a cerebellum and primitive basal ganglia, which contain body maps, as well as the vestibular reward mechanism. So the principle of external and internal guidance of reward based goal directed action also applies equally to fish and humans. Such principles also apply to all other classes of vertebrate, including birds and mammals, examples for whom beat induction behavior has been provided (Schachner et al., 2009; Fitch, 2013).

If this still sounds farfetched it is worth here quoting Darwin on the origin of music (Darwin, 1871). His theory was that music must have evolved from reproductive vocal behavior. “Although the sounds emitted by animals of all kinds serve many purposes, a strong case can be made out, that the vocal organs were primarily used and perfected in relation to the propagation of the species” [p. 875]. On reviewing the vocal behavior of frogs, toads, tortoises, alligators, birds, mice, and gibbons, he concludes: “Unless females were able to appreciate such sounds and were excited or charmed by them, the persevering efforts of the males, …, would be useless; and this is impossible to believe” [p. 878] After reviewing human music of different cultures, he reaches similar conclusions: “All these facts with respect to music and impassioned speech become intelligible to a certain extent, if we may assume that musical tones and rhythm were used by our half-human ancestors, during the season of courtship.…The impassioned orator, bard, or musician, when with his varied tones and cadences he excites the strongest emotions in his hearers, little suspects that he uses the same means by which his half-human ancestors long ago aroused each other's ardent passions during their courtship and rivalry” [p. 881].

## 4. Discussion and future directions

In the above we have presented in some detail the outline of a new synthesis of vestibular and rhythm research. As well as accounting for the neurobiological aspects of rhythm perception, and to some extent answering the questions posed by Zatorre et al. (2007), the new synthesis provides an explanation for a number of phenomena not often considered by rhythm researchers. Below we discuss these and outline some new directions for both vestibular and rhythm research.

### 4.1. Oscillator vs. somatotopic explanations of beat induction

The first point we should emphasize is that the above account of beat induction is fundamentally different from the oscillator theory. It proposes that a detected beat, as a motion in the somatotopic body maps of a lateral sensory-motor circuit, rapidly transfers to become a “felt” beat as a motion in the somatotopic body maps of a medial circuit. The particular motion in the maps most likely involves a head region and within the basal ganglia a selected habitual motion such as head bobbing. The SNr only contains a motor map of the head region which is primarily activated by passive or active head motion. It will thus be experienced as a self-motion, i.e., as a kind of internal dance. The fundamental difference here with the oscillator approach is that there is no need to propose clocks or oscillators in the BG, as the internal motion associated with a beat is distributed throughout the somatotopic maps which constitute the totality of the sensory-motor circuits activated. As these circuits accurately represent the dynamics of the body they are able to accurately model a repetitive body motion synchronized with a rhythmic input. This also explains the existence region for beat induction, since only motions close to the natural frequencies of the body will be selected.

According to this theory, increased activity in the SMA and BG associated with a beat does not represent the activation of a clock in the BG, but is rather a sign that the body motion has transferred to the medial circuits, i.e., that the motor maps in the SMA/CMAc and putamen have become more active. Conversely, the fact that activity in STG is not increased in a beat condition does not mean that it is not active. The medial sensorimotor circuits still have access to the modulation power spectrum representing the stimulus input in STG. Reciprocal connectivity between the SMA/CMAc and STG thus allows the medial circuits to have an external reference.

The deficit that patients suffering from Parkinson's disease (PD) show in beat induction has been cited as evidence for a clock or oscillator in the BG (Grahn and Rowe, 2009). From the somatotopic and vestibular perspective the symptoms of PD can be alternatively explained in terms of disorganization of the somatotopy (Nambu, 2011). It is thought that one of the roles of dopamine in the BG is to maintain the detailed specificity of body parts in the somatotopic maps. In PD the different body parts lose their specificity so that motor programmes experience cross-talk or interference. A similar pathophysiology can be ascribed to dystonia and other movement disorders (Nambu, 2011). Thus, the beat induction deficit shown in PD may be explained as a disruption to the body map so that a patient cannot experience the habitual self-motion normally associated with a beat.

A second important point we should emphasize is that the existence of two distinct networks for beat “finding” and beat “feeling” creates a fundamental problem for the modelers who argue that the basal ganglia are the locus of oscillations underlying rhythm and time perception (e.g., Matell and Meck, 2004). The problem is that if the BG is involved with beat “feeling” but not “finding,” any such BG based oscillators cannot be involved in periodicity detection. Thus, somewhere else in the brain is detecting a beat and handing it over to the BG oscillators who get the period and phase for free. If the oscillator networks are not involved in period detection then their role is simply to keep or “feel” the beat. This then raises the question of what possible evolutionary purpose is served by such a mechanism? It is difficult to envisage either a natural or sexual selective pressure to evolve mechanisms that are both lazy (they don't do beat detection) and disembodied (there is no reference to the body).

In contrast a mechanism involving two distinct sensory-motor circuits, one attention dependent, and the other reflexive, can be seen, as we have described, to be the reflection of systems evolved to manage posture, gaze, and autonomic function during locomotion and which have been shaped by both natural and sexual selection. The comparative anatomy and physiology can be traced with some precision to implicate the origin of the neurobiological mechanisms to those of a common ancestor of anamniotes for whom vestibularly mediated reproductive behavior was central to their existence. This account thus provides for a proper account for the motive or drive for beat induction and it does so within an evolutionary perspective which is consistent with Darwinist views of the origin of music.

### 4.2. Why do humans dance?

We may also apply this approach to the question of why humans dance. It is well-accepted that many species of birds exhibit dance behavior during courtship. These include, for example, birds of paradise, the Japanese crane, and the Western grebe, to name a few, but even many species of gull engage in courtship dance. Evidence can also be presented from other classes of vertebrate, including fish. Although such mating behaviors are almost universal, some authors have argued that only humans exhibit true “entrainment.” For example, Merchant and Honing (2014) have argued that only humans can entrain to auditory rhythms because of the poor connectivity in the primate brain from auditory cortex to SMA compared to the human brain. We believe this approach to be fundamentally in error for at least two reasons. First, in line with the oscillator theories of beat induction, it focuses exclusively on the medial sensory-motor basal ganglia circuits, to the exclusion of the lateral PMC cerebellar circuits. It thus cannot be an account of beat finding, which makes use of a lateral system (Grahn and Rowe, 2013). Further, as noted above, there is now evidence that some non-human species also exhibit beat induction behavior (Bregman et al., 2012).

A second reason is that such basal ganglia centric theories ignore another fundamental difference between human and primate, which is that humans are bipedal. The dynamical properties of the primate vs. human body maps in the sensory-motor circuits will also share this fundamental difference. The human bipedal body has eigenmodes which the knuckle walking tetrapod does not, and this will be reflected in the body representations in the brain. One can also apply the eigenmode analysis to other species, e.g., birds are essentially bipedal, horses have pendular long necks and fish have bilateral symmetry.

Quite apart from the issue of entrainment though, the oscillator theories have nothing to say about dancing to music which does not have a beat. On this we point we also fundamentally disagree with Honing (2012) that “without it there can be no music.” In fact a large proportion of the world's music falls into this category, including music with ametrical rhythms, such as recitative, plainsong or more modern ambient styles, or much music of the Romantic era, which employs a deep rubato. One would not tap the foot or nod the head in these cases, but one might though sway the whole body along with the larger scale motions associated with phrasing, and such swaying is also a form of vestibular self-stimulation, as is chanting. Again it is possible to associate such gestural or whole body self-motion perceptions with somatotopic maps within the medial sensory-motor circuits as a kind of “inner movement” in the sense of Truslit's *innere Bewegtheit*. However, in the same way that sounds which are above vestibular threshold for beat based music may have a direct input, so may it be for very expressive music, especially if it highly dynamic, which often is the case in some of the most passionate passages in the music of Chopin (Todd, 1992a). Indeed, one might say, again in the Truslitian spirit, that the aim of the performer is to create and communicate the sense of motion by means of the temporal and dynamical shaping of phrases in motional expression or *Bewegungsvorgänge*. From a neurobiological perspective such motional percepts may almost literally be created as was predicted by Truslit, i.e., by either indirect associative links of sound shapes to vestibular centers in the body maps or by direct vestibular activation of body maps by sound above the vestibular threshold.

### 4.3. Why is human dance music so loud?

This last issue leads us to the question of why human dance music has a tendency to be so loud. One of course thinks of raves and discos, but also live performances of popular music styles, such as rock, metal, punk, reggae, etc. Such music also tends to be very bassy, often including infrasound. What all of these have in common is that they make use of amplification, available in human culture since the 1950s. Loud dance music is not, however, dependent on amplification. There are many examples of loud drum music, such as Japanese taiko, which can be traced to antiquity. Many examples can also be found in the classical orchestral repertoire, such as Stravinsky's *Le Sacre du Printemps*. Concern about hearing loss from loud music is not limited to rock musicians but is an occupational hazard also of orchestral musicians.

Again here the oscillator theories fall silent. In contrast the sensory-motor theory provides a natural explanation based on reward produced by acoustic activation of the vestibular system and the powerful immediate drive to dance from the vestibular reflexes (Todd and Cody, 2000; Todd, 2001). The bassy aspect of such music can be attributed to the low-frequency sensitivity of the vestibular apparatus (Todd et al., 2000a, 2008b). It also gives an account of the association of loud amplified music with dance drugs, since the hedonic gain of the vestibular reward mechanism is amplified by the action of such drugs on the mesolimbic dopamine system. In such environments it is not even necessary to have a normally functioning cochlea, as testified by the fact that many within the deaf community enjoy dancing, especially when they can feel the acoustic vibrations. They do though require, we suggest, an intact vestibular system.

Recent experiments have confirmed that loud bass plays an important role in synchronizing human dance (van Dyck et al., 2013). An important fMRI experiment that needs to be done to clarify the role of loud sounds is to investigate “quiet” vs. “loud” beat induction, checking by means of VEMPs that the “quiet” beat induction stimuli did not activate the vestibular system. To be absolutely sure that acoustic activation of the vestibular system was not a source of contamination it would also be necessary to run a similar experiment with avestibular patients who one could be sure had no vestibular response to sound.

### 4.4. Future perspectives—rhythm and balance, two sides of the same coin

If it is accepted that rhythm perception is a sensory-motor phenomenon, from the perspective of the new synthesis, rhythm and vestibular perception and cognition should become just two sides of one field of enquiry where the research techniques of one can lend to the other. Thus, experimental methods which have been developed for rhythm research, such as psychophysical or electrophysiological procedures should be adaptable as tests of central vestibular function and cognition. Conversely methods developed for vestibular research should also be applicable to rhythm perception and production.

This applies equally to the study of normally functioning individuals as to the categories of patients that are considered in more clinical or neuropsychological studies, so that for example, patients with rhythm deficits may well have also vestibular dysfunction, and vice-versa. Thus, as well as using vestibular patients to check for the possibility of vestibular contamination in rhythm experiments, as above, there are many interesting experiments with rhythm that could be used as a test for vestibular cognitive deficits. As well as avestibular patients or patients with unilateral vestibular loss, hypervestibular patients, such as those with superior canal dehiscence (SCD) could also be an interesting group to study, as noted already by Truslit in his reference to the work of Tullio (1929) and Watson et al. (2000). The somatotopic viewpoint could also act as a spur to more refined experiments to look for activation in specific motor maps and body parts in high resolution imaging studies. Thus, for example, it would be interesting to know not just that the motor areas of the basal ganglia are active in a beat induction task, but is the activity confined to the SMA map, and which part of the map—head, hand, or toe? Are there correlations between the body part activations in PMC, SMA, and putamen, for example? Topographic organization could be investigated by including active as well as passive listening tasks, requiring subjects to actually move the parts or imagine the movement of different body parts.

The somatotopic viewpoint should also inspire new computational models of beat induction. As we noted above, models which are limited to only a periodicity analysis, whether by utilizing the power spectrum or by autocorrelation, cannot provide an account of beat induction since they contain no concept of the existence region of a beat. Only by combining such sensory representation along with externally vs. internally oriented sensory-motor representations can a beat be adequately modeled in a manner consistent with the experimental evidence. Since a beat in music is often intended for dancing to, ultimately what is required is a computational model which simulates dancing. The construction of a dancing robot with its own auditory and vestibular systems, or at least a simulation of one, would be one way to achieve this. Such dancing systems already exist, e.g., the Adelbaran Robotics NAO humanoid robot, and these could be harnessed for the purpose of rhythm/vestibular research since a lot can be learned from a cybernetic approach. Their dance routines, although quite sophisticated, are not spontaneous but make use of preprogrammed movement sequences to simple beat rhythms, perhaps not unlike habitual over-learned routines in the basal ganglia. In addition to the preprogrammed sequences, however, NAO's brain must have a representation of the dynamic and kinematic properties of its body as a feed-forward model in order provide the appropriate motor commands to achieve the movement goal. NAO also needs a mechanism to maintain balance, i.e., some kind of electronic vestibular system.

We should also broaden horizons within the domain of comparative neuroanatomy and physiology. As noted above, there are many examples of dance in non-human and non-mammalian species. The role and function of vestibular self-stimulation in animal and human behavior should be of interest to both vestibular and rhythm researchers. We have much to learn from fish chorusing, for example. There are also many examples of dance like behavior in invertebrate species, including some very surprising ones.

## 5. Concluding remarks

In this paper we have reviewed the history of the sensory-motor theory of rhythm and beat induction since it first appeared in the literature some 20 years ago. There have been remarkable developments particularly in human neuroimaging studies in the last 15 years which have essentially confirmed the basic hypothesis, i.e., that rhythm and beat perception is mediated by an extensive and distributed network which includes both sensory and motor representations. We strongly believe that further significant progress will come in the next 10 years when the somatotopic internal body motion approach is fully realized and the link between vestibular and rhythm research is developed. Only such an approach, we believe, can provide explanations which are fully consistent with the Darwinian theory of evolution.

### Conflict of interest statement

The authors declare that the research was conducted in the absence of any commercial or financial relationships that could be construed as a potential conflict of interest.
